# Genetic basis of drought tolerance during seed germination in barley

**DOI:** 10.1371/journal.pone.0206682

**Published:** 2018-11-02

**Authors:** Samar G. Thabet, Yasser S. Moursi, Mohamed A. Karam, Andreas Graner, Ahmad M. Alqudah

**Affiliations:** 1 Department of Botany, Faculty of Science, University of Fayoum, Fayoum, Egypt; 2 Research Group Genome Diversity, Leibniz Institute of Plant Genetics and Crop Plant Research, OT, Germany; 3 Research Group Resources Genetics and Reproduction, Department Genebank, Leibniz Institute of Plant Genetics and Crop Plant Research (IPK), OT, Germany; Julius Kühn-Institut, GERMANY

## Abstract

Drought is one of the harshest abiotic stresses hindering seed germination, plant growth, and crop productivity. A high rate and uniformity of germination under stressful conditions are vital for crop establishment and growth; thus, for productivity. A better understanding of the genetic architecture of seed germination under drought stress is a prerequisite for further increasing yield potential. Barley is considered one of the most abiotic stresses-tolerant cereals. Elucidating the drought tolerance of barley during seed germination would indeed pave the way towards improving the performance of all cereals. However, we still know relatively little about the genetic control of drought tolerance during the seed germination phase. In our study, 218 worldwide spring barley accessions were subjected to PEG-induced drought during seed germination. Induced drought stress "20% PEG" significantly reduced the seed germination parameters and seedling related traits. A genome-wide association scan (GWAS) was used to identify genomic regions associated with our trait of interest. In total, 338 single nucleotide polymorphisms (SNPs) were found to be associated with several traits distributed across seven barley chromosomes, of which 26 genomic regions were associated with candidate genes. The current study found some of the quantitative trait loci (QTL) that have previously been reported to be linked to seed germination-related traits under drought conditions, as well as some new associations. Noteworthy, the identified QTL colocalized with a number of genes (within interval ±0.5 Mbp) that are exclusively distributed on chromosomes 1H, 2H, and 5H. The annotation of these genes in barley shows their roles in drought tolerance through encoding different transcription factors. The function of the identified genes during seed germination was also confirmed by the annotation of their counterparts in *Arabidopsis*. The current analyses show the power of the GWAS both for identifying putative candidate genes and for improving plant adaptive traits in barley.

## Introduction

Barley (*Hordeum vulgare* L.) is considered to be the fourth most important cereal crop worldwide [1], largely due to its exceptional adaptations towards growing in a variety of different environmental conditions. Barley is particularly able to adapt to diverse conditions, such as drought and salt compared to other cereals [2]. Such a useful characteristic allows barley to grow in regions where other cereals for instance wheat cannot grow well.

Abiotic stress conditions are a widespread problem worldwide, whereas drought is the most important factor limiting crop growth and is becoming more common, particularly in arid and semiarid regions [3]. The impact of drought stress is obvious on plant performance and yield; the germination and seedling development phases being the period most sensitive to such conditions in barley [4] as well as in the most other crops [5]. Drought stress at a very early developmental stage delays seed germination and reduces the rate of germination [4,6]. Therefore, understanding natural variation and genetic control of germination and related traits under drought stress can help to improve barley crop growth and yield.

Barley is an excellent model cereal crop for studying the genetics of developmental and adaptive traits as it is known for its high degree of genetic diversity with regard to stress tolerance [4]. Evaluation of barley genotypes for yield stability revealed high genetic plasticity under drought stress conditions [7,8]. Barley uses sophisticated strategies in response to drought stress, having evolved different mechanisms to alleviate the detrimental effects of harsh environments by altering its physiological, molecular, and cellular functions. For instance, molecular mechanisms of the plant used for abiotic stress response have allowed us to develop stress-tolerant cultivars [9,10].

Genetically, drought tolerance-associated traits are quantitative traits, whereby many genes are involved with minor effect [11,12]. Several studies have been carried out to study the genetic factors underlying drought-related traits in barley, revealing that most of the detected QTL control developmental and adaptive traits in addition to drought tolerance [13–16]. Indeed, characterization of stress-related genes was preceded by the isolation of candidate genes and gene expression to study drought stress response [17]. These strategies enabled researchers to identify key stress regulators by deriving regulatory networks through the application of different “omics” (transcriptomics, metabolomics, and proteomics) approaches [18]. Sequence information on complete genomes of model plants and several crop species have significantly enhanced our ability to identify genes associated with drought tolerance. Thus, the knowledge gained from model plants can be extrapolated to improve stress tolerance in other crop species.

Improving yield is always the primary goal of breeding. A plethora of studies has been conducted to determine the quantitative traits related to drought tolerance at a productive phase in barley. For example, QTL analyses to understand the genetic basis of yield and yield-related traits were made in barley, including kernel weight, a number of grains per spike [19,20], grain yield [20,21] grain weight, spike length [22], spike morphology, and grain yield [23].

Genome-wide association scan (GWAS) analysis is an advanced approach used to understand the genetics of natural variation in the trait of interest in a plant. This approach was used to study yield, yield components, developmental, and physiological and anatomical traits in a diverse barley collection under drought stress [24]. However, little is yet known about the genetic variations underlying seed germination and related adaptive traits in barley under drought stress.

The present study, therefore, aims at studying the genetic variation of seed germination related-traits under artificially induced-drought stress conditions in order to identify the genetic factors controlling the variation in these traits. This study contributes to understanding the genetic control of natural variation in drought tolerance-related traits on which further genetic analysis can be built. Here, we found new QTLs associated with the natural variation of seed germination-related traits in addition to many previously detected QTLs. Our QTL analysis was extended to identify genes colocalized with the detected QTL which underlie our traits of interest.

## Materials and methods

A collection of 218 worldwide spring barley accessions was used in the current study. The collection includes 149 cultivars, 57 landraces,and 18 breeding lines. The origins of these accessions were from Europe (EU, 108), West Asia and North Africa (WANA, 45), East Asia (EA, 36),and the Americas (AM, 29). More information about the population structure has been published by [25–27].

### Germination test

For the germination test, only ten seeds per genotype were randomly selected for each of three of the replications. The seeds were placed on wetted filter paper in 9-cm-diameter Petri dishes in order to evaluate the growth performance and phenotypic variation in seedling related traits among genotypes. Drought stress was induced by adding PEG-6000 at a concentration of 20% (w/v), while distilled water without PEG was used as a control (unstressed seeds). The Petri dishes were placed in an incubator at 20°C in the darkness. The experiment was conceived as a randomized complete block design (RCBD). The seeds were considered as germinated when the radicle reached at least 2 mm in length. Germination was scored at 24 hours intervals for 12 consecutive days. In total, twenty-two germination and growth performance-related traits were scored as explain in Table 1.

**Table 1:** The name and abbreviation of measured traits and respective description of measurements.

**Trait**	**Name**	**Measurement description**
G%	Germination percentage	(G%) $=\frac{n}{N}$ ×100, n is the number of germinated seeds at the end of experiment, N is the total number of seeds.
GP	Germination Pace	(GP) $=\frac{N}{\sum \left(n\times g\right)}\times 100$, N is the number of germinated seeds at the end of experiment, n is the number of newly germinated seed at certain day g, g = (1, 2, 3….)
DTI (G%)	Drought tolerance index (germination percentage)	DTI $(G\%)=\frac{G\%\;\mathrm{under}\;\mathrm{drought}}{G\%\;\mathrm{under}\;\mathrm{control}}\times 100$
DTI (GP)	Drought tolerance index (germination pace)	DTI $(GP)=\frac{\mathrm{GP}\;\mathrm{under}\;\mathrm{drought}}{\mathrm{GP}\;\mathrm{under}\;\mathrm{control}}\times 100$
Reduction _ G%	Reduction _ Germination percentage	Reduction of G% = G% under control–G% under drought
Reduction _ GP	Reduction _ Germination Pace	Reduction of GP = GP under control–GP under drought
SL	Shoot Length	Shoot length was measured by a scaled ruler (in cm)
Reduction _SL	Reduction _Shoot Length	Reduction of SL = SL under control–SL under drought
SL_DTI	Shoot Length_ Drought tolerance index	SL_DTI $=\frac{\mathrm{SL}\;\mathrm{under}\;\mathrm{drought}}{\mathrm{SL}\;\mathrm{under}\;\mathrm{control}}\times 100$
RL	Root Length	Root length was measured a scaled ruler (in cm)
Reduction _RL	Reduction _Root Length	Reduction of RL = RL under control–RL under drought
RL_ DTI	Root Length_ Drought tolerance index	RL_DTI $=\frac{\mathrm{RL}\;\mathrm{under}\;\mathrm{drought}}{\mathrm{RL}\;\mathrm{under}\;\mathrm{control}}\times 100$
SRR	Shoot Length /Root Length Ratio	as the ratio of the SL to the RL
FW	Fresh Weight	Fresh weight was recorded in (g) using a sensitive balance (Sartorius AC 1215, Germany)

Germination parameters were assessed according to International Seed Testing Association rules (ISTA) as follows:

Germination percentage is expressed as (G%) $G\%=\frac{n}{N}\times 100$

Where n is the number of germinated seeds at the end of the experiment and N is the total number of total sown seeds.

Germination pace is expressed as (GP)

$$GP=\frac{N}{\sum \left(n\times g\right)}\times 100,$$

Where N is the total number of germinated seeds at the end of the experiment and n is the number of germinated seeds on day g (1, 2, 3,..).

The drought tolerance index (DTI) was calculated for G% and GP, according to the equations:

$$DTI\;(G\%)=\frac{G\%\;\mathrm{under}\;\mathrm{drought}}{G\%\;\mathrm{under}\;\mathrm{control}}\times 100$$

$$DTI\;(GP)=\frac{\mathrm{GP}\;\mathrm{under}\;\mathrm{drought}}{\mathrm{GP}\;\mathrm{under}\;\mathrm{control}}\times 100$$

The reduction of G% and GP were also calculated as follows:

ReductionofG%=G%undercontrol–G%underdrought

ReductionofGP=GPundercontrol–GPunderdrought

For Shoot length (SL) and root length (RL) measurement, 10 seeds were grown in rolling paper [28], the paper rolls were placed in 1L beakers half filled with water for control and 20% PEG for drought. After 12 days, SL and RL (in cm) were manually measured using a scaled ruler. The fresh weight (FW) of seedlings was recorded (g) using a sensitive balance (Sartorius AC 1215, Germany). The shoot-root ratio (SRR) was calculated as the ratio of the SL to the RL. Reduction of SL, RL, and FW were calculated as follows:

ReductionofSL=SLundercontrol–SLunderdrought

ReductionofRL=RLundercontrol–RLunderdrought

Also, the drought tolerance index (DTI) was calculated for SL and RL:

$$SL\_DTI=\frac{\mathrm{SL}\;\mathrm{under}\;\mathrm{drought}}{\mathrm{SL}\;\mathrm{under}\;\mathrm{control}}\times 100$$

$$RL\_DTI=\frac{\mathrm{RL}\;\mathrm{under}\;\mathrm{drought}}{\mathrm{RL}\;\mathrm{under}\;\mathrm{control}}\times 100$$

### Data analyses

Analysis of variance (ANOVA) was conducted for germination and growth performance-related traits to compare controlled and induced-drought stress conditions using GENSTAT (2015) [29] for Windows Ver. 17 (VSN International, Hemel Hempstead, UK). Broad-sense heritability (*H*^*2*^) was estimated for the studied traits under both conditions separately using GENSTAT 17.

Broad-sense heritability (*H*^*2*^) = σ^2^g / (σ^2^g + σ^2^g × t/e + σ^2^e/re)

Where σ^2^ g is genotypes variance; σ^2^ g.t is the variance of the interaction genotype × treatment, r is replicates, and e the error.

Residual Maximum Likelihood (REML) was used to analyze the phenotypic data of 193 accessions, while the Best Linear Unbiased Estimates (BLUEs) were calculated to estimate the mean of each trait for each accession under each treatment using GENSTAT 17.

### Marker-trait association analysis (GWAS)

The accessions of the collection were genotyped with a high-density 9K SNPs chip from Illumina^TM^. This chip assayed 7842 SNPs and was first described by Comadran et al. [30] We used only the markers which have minor allele frequency (MAF) ≥ 0.05 (6355 SNP) with their POPSEQ genetic positions [31]. This chip was used to study the developmental traits in spring barley and more data on it can be found in [25–27]. Using the estimated phenotypic traits (BLUEs) and genotypic data, a mixed linear model (MLM) was performed to determine marker-trait association using GenStat 17 (Genstat, 2015). A kinship matrix was used as a correction of population structure to control the false positive associations (Genstat, 2015). Markers with a threshold P-value of 0.001 (i.e.,–log10 P ≥ 3) were considered as significant associations and were used in another test of robustness, i.e. false discovery rate (FDR) at 0.01 [32]. The SNPs exceeding FDR were considered as true associations that were used in annotation the candidate gene; however, we still consider the markers with -log_10_ P-values ≥3 in the highly associated regions to see the effect of these regions on the traits. We used the recent barley genome dataset and geneset [33] (BARLEX; http://apex.ipk-gatersleben.de) to annotate the genes that could be considered as candidates for significant associations, i.e. those markers which show a consistent effect on traits and highly associated with SNPs passing ≥ FDR using their physical position. The candidate genes, which are very close to, the associated SNPs within 0.5 Mbp (~ 0.1 cM) were considered as highly putative candidate genes. Functional annotations of the candidates were also confirmed using already known information about other plants, such as Arabidopsis.

## Results

### Traits natural phenotypic variations

**Fig 1:**
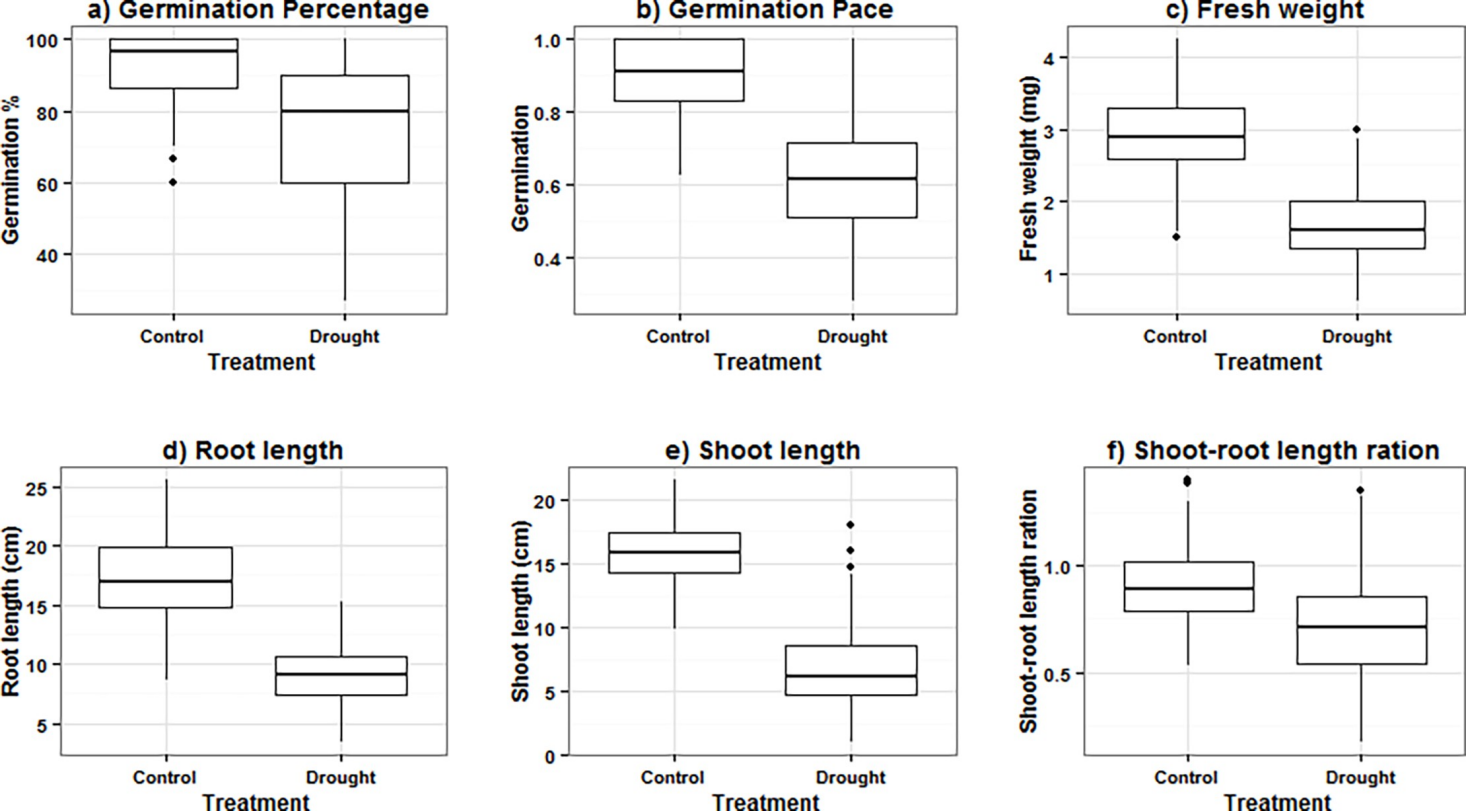
Boxplot analysis of variation of the traits; a) Germination percentage, b) Germination pace, c) Fresh weight, d) Root length, e) Shoot length and f) Shoot-root length ration in barley genotypes under control and drought stress (20% PEG).

**Fig 2:**
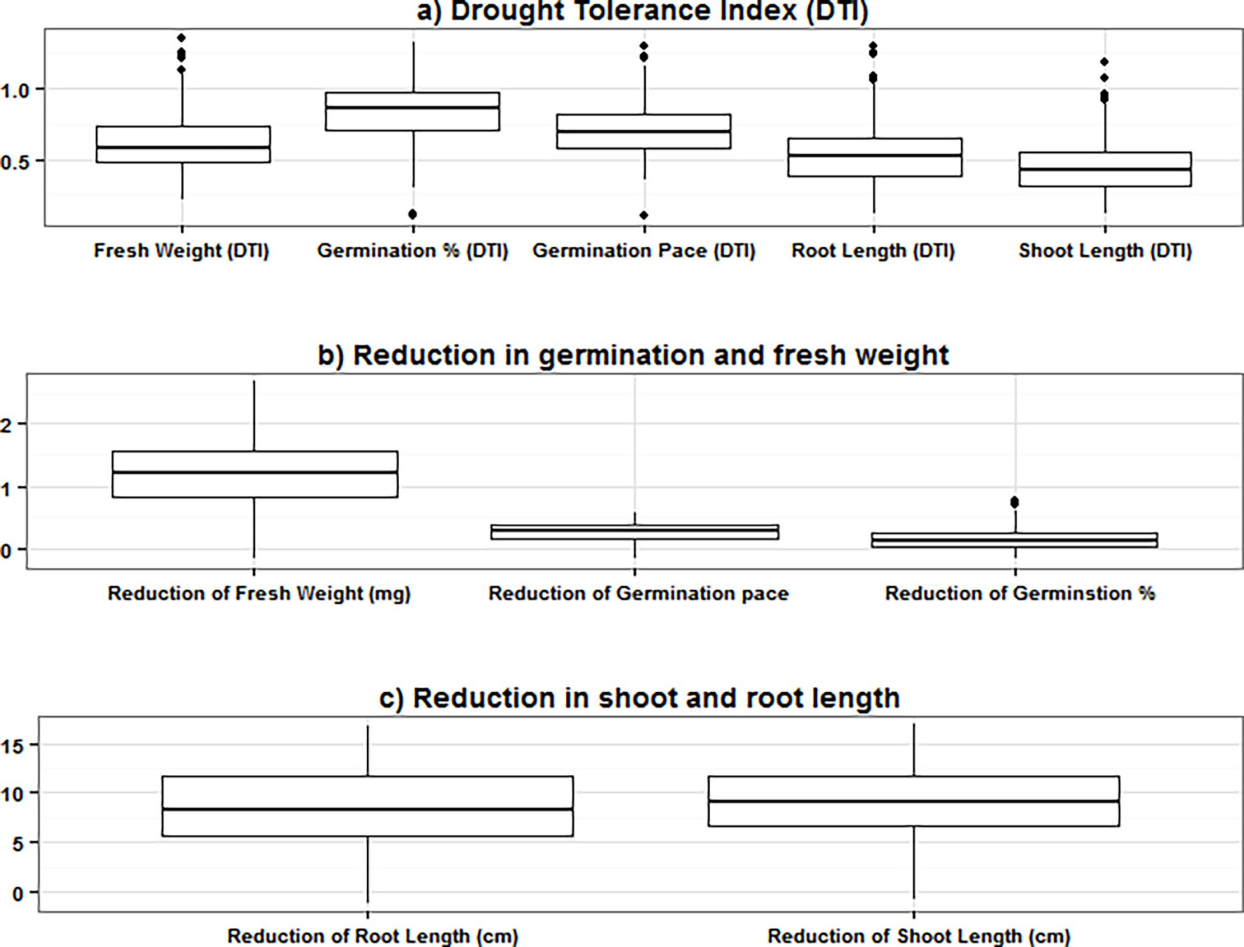
Boxplot analysis of variation of the traits; a) Drought tolerance index, b) Reduction in germination and fresh weight, c) Reduction in the shoot and root length in barley genotypes under control and drought stress (20% PEG).

A large phenotypic variation for all traits was found among the genotypes under control conditions as well as under induced drought (Figs 1 and 2). The genotypes revealed a highly significant variation (*P* ≤ 0.001) under control and induced drought. Heritability and genetic variance estimated from ANOVA are summarized in Table 2. Finally, 193 accessions passed the germination test and were used in the final analysis.

**Table 2:** Heritability and analysis of variance for 193 barley genotypes under control and drought stress conditions for all traits.

Trait	ANOVA^l^				ANOVA			
	T	G	T × G	*H*^*2*^	T	G	T x G	*H*^*2*^
**Germination %**	***	***	***	0.95	***	***	***	0.95
**Germination pace**	***	***	***	0.85	***	***	***	0.91
**Shoot Length (cm)**	***	***	***	0.93	***	***	***	0.95
**Root Length (cm)**	***	***	***	0.95	***	***	***	0.93
**Fresh Weight (mg)**	***	***	***	0.93	***	***	***	0.94
**SL/RL^a^**	***	***	***	0.91	***	***	***	0.95
**G%_DTI^b^**	—	***	—	0.92				
**GP_DTI^c^**	—	***	—	0.87				
**SL_DTI^d^**	—	***	—	0.94				
**RL_DTI^e^**	—	***	—	0.98				
**FW_DTI^f^**	—	***	—	0.90				
**Reduction_G%^g^**	—	***	—	0.91				
**Reduction_GP^h^**	—	***	—	0.83				
**Reduction_SL^i^**	—	***	—	0.94				
**Reduction_RL^j^**	—	***	—	0.94				
**Reduction_FW^k^**	—	***	—	0.86				

^a^SL/RL—Shoot Length_Root Length Ratio

^b^G%_DTI—Germination percentage_Drought Tolerance Index

^c^GP_DTI—Germination Pace_Drought Tolerance Index

^d^SL_DTI—Shoot Length_ Drought Tolerance Index

^e^RL_DTI—Root Length_ Drought Tolerance Index

^f^FW_DTI—Fresh Weight_ Drought Tolerance Index

^g^Reduction_G%—Reduction_ Germination percentage

^h^Reduction _ GP—Reduction_ Germination Pace

^i^Reduction_SL—Reduction_Shoot Length

^j^Reduction_RL—Reduction_ Root Length

^k^Reduction_FW—Reduction_Fresh Weight.

^l^Significance of the sources of variability:G denotes genotypes and T for treatments whrease *h*^*2*^ is the broad- sense heritability*, **, *** Significant at P ≤ 0.05, P ≤ 0.01 and P ≤ 0.001 level of significance, respectively.

There was a considerable reduction in performance for all traits compared to control under induced drought stress (20% PEG), although some genotypes revealed a G% of up to 100%, and GP up to 1. Notably, for some traits, a set of genotypes showed **a** better performance under induced drought than under controlled conditions (S1 Table).

Under control conditions, the genotypes’ mean values were 87%, 0.88, 15.73, 16.84, 1.01 and 2.92 for G%, GP, SL, RL, SL/RL, and FW, respectively (Fig 1 and S1 Table). Under induced drought stress (20% PEG), the genotypes’ mean values were 72%, 0.63, 6.8, 9, 0.77 and 1.79 for G%, GP, SL, RL, SL/RL and FW, respectively (Fig 1 and S1 Table). Heritability ranged from 0.83 to 0.98 under control conditions and from 0.91 to 0.95 under stress conditions (Table 2).

A large variation among genotypes was detected with regard to their responses to induced drought stress. For DTI, the genotypes mean values were 84.50, 71.82, 43.1, 58 and 61.7 for G%, GP, SL, RL, and, FW, respectively (Fig 2 and S1 Table). For reduction, the genotypes mean values were 15%, 0.24, 8.9, 7.9 and 1.14 for G%, GP, SL, RL, and FW, respectively (Fig 2 and S1 Table). The heritability varied from 0.87 to 0.98 for GP_DTI and RL_DTI, respectively (Fig 2 and S1 Table). Likewise, the heritability for reduction parameters ranged from 0.83 to 0.95 for Reduction_GP and Reduction_RL, respectively (Fig 2 and S1 Table). These data suggest that drought stress has a clear impact on seed germination and growth other performance-related traits in barley. The wide range of phenotypic variation along with high heritability values observed represented a solid basis for the genetic dissection of individual traits by genome-wide association analysis.

### Correlations analysis

**Fig 3:**
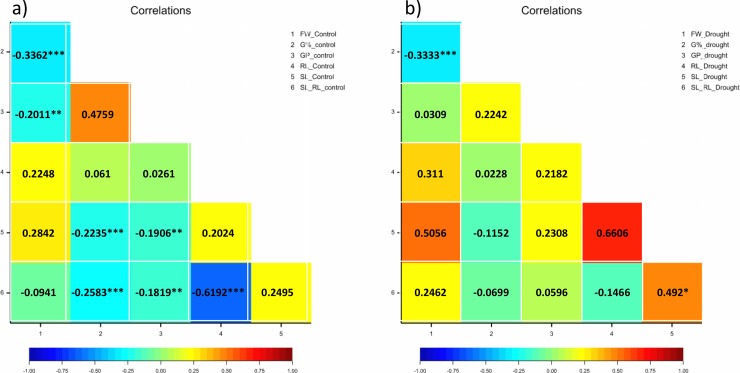
**Correlations of the studied traits in barley genotypes (a) under control and (b) drought stress (20% PEG).** The degree of significance indicated as *P, 0.05; **P, 0.01; ***P, 0.001.

Pearson’s phenotypic correlations between the genotypes under each treatment condition show differences among them in response to drought stress. Under control conditions, the positive correlations were non-significant, while the negative correlations were significant except between FW and SL_RL. For example, FW showed a negative and significant correlation with G% and GP (r = -0.33*** and -0.20**, respectively). Also, G% showed significant and negative correlations with SL and SL_RL (r = -0.22*** and -0.25***, respectively). Likewise, GP significantly and negatively correlates with FW, SL and SL_RL r = -0.20**, r = -0.19**and -0.18**, respectively (Fig 3A).

Under induced drought, there are positive correlations between studied traits,e.g. RL_drought and SL_ RL_drought (r = 0.492*). The negative correlations were significant between FW_drought and G%_drought, r = -0.33*** (Fig 3B). These findings indicate that the drought-tolerant accessions showed a good growth performance (RL, SL and their ratio) at early developmental stages.

### Natural genetic variation for studied traits by GWAS analysis

GWAS analysis of the 193 accessions used in the current study was performed to discover the natural genetic variation of the studied traits. We detected a total of 338 significant marker-trait associations with –log_10_ p-value ≥3 of which 111 SNPs passing FDR test (S2 Table) distributed over the barley chromosome seven.

The physical positions of these significant SNPs were used to find the candidate genes, which are collocated or very close to them (around 0.5 Mbp ~ 0.1 cM). The candidates which showed consistency (several markers in the same position exceeding FDR) were distributed exclusively on chromosomes 1H, 2H, and 5H and classified into two categories (i) Adaptive genes; control-specific, i.e. genes which regulate trait variation under control only, or drought-specific, i.e. genes which regulate trait variation under induced drought only, (ii) Constitutive genes, i.e. genes which regulate trait variation under both control and drought conditions.

#### Germination percentage

In total, 104 SNPs showed association (with–log_10_ p-value ≥3) with germination percentage parameters. The distribution of SNPs for each individual trait is outlined in Fig 2 and Panel A in S1 Fig summarizes the results for all traits. For G%, 11 SNPs were detected on chromosomes 1, 2, 4, 5 and 7 under control. The highest effects were detected on chromosomes 2 (–log_10_ p-value = 4.15), 5 (4.73) and 7 (4.88) at 57cM, 80.8cM, and 109.7cM, respectively. Twenty-one SNPs were identified on chromosomes 1, 2, 3, 4, 5 and 7 under induced drought. The most significant one (with–log_10_ p-value = 8.54) was observed on chromosome 4 at 111.1cM. For G%-DTI, 33 SNPs were mapped on chromosomes 1, 2, 3, 4, 5 and 7. The highest effects (–log_10_ p-value = 6.05 and 6.11, respectively) were observed on chromosomes 3 and 4 at 90.2cM, and 34.6 cM, respectively. Thirty-nine SNPs were detected on chromosomes 1 to 7 for Reduction_ G%. The most significant one (with–log_10_ p-value = 6.65) was observed on chromosome 5 at 44.2cM. Of these, 23 SNPs showed an association (several markers in the same position) with candidate genes. Twenty-two genes are constitutive genes for G%-DTI and reduction of G%, and one drought-specific (*HORVU1Hr1G048400*) for G%_drought (Table 3 and S3 Table). No genes were identified for G% under control (Table 3 and S3 Table).

**Table 3:** The functional annotation of the putative candidate genes associated with the estimated traits under drought and control growth conditions.

Trait	Chr^o^	Pos^p^	iSelect Marker	SNP^q^ Pos	Barley gene (2017)	start	end	Annotation in barley
**RL_drought^a^**	1	46.8	BOPA2_12_11498	31354146	HORVU1Hr1G012490	31356072	31359183	E3 ubiquitin-protein ligase SINA-like 11
**Reduction_SL^b^**	1	47.8	BOPA2_12_30243	389343900	HORVU1Hr1G052560	3.89E+08	3.89E+08	DDB1- and CUL4-associated factor 8
**G%_DTI^c^**	1	47.9	SCRI_RS_130590	353968495	HORVU1Hr1G047820	3.54E+08	3.54E+08	Aldo-keto reductase family 4 member C9
**G%_DTI**	1	47.9	SCRI_RS_219043	358992876	HORVU1Hr1G048450	3.59E+08	3.59E+08	actin depolymerizing factor 6
**RL_drought**	1	48.1	SCRI_RS_149971	103520348	HORVU1Hr1G023460	1.04E+08	1.04E+08	gibberellin 2-oxidase 6
**Reduction_G%^d^**	1	48.1	SCRI_RS_7813	352312914	HORVU1Hr1G047690	3.52E+08	3.52E+08	Tubulin-specific chaperone D
**G%_DTI**	1	48.1	SCRI_RS_7813	352312914	HORVU1Hr1G047690	3.52E+08	3.52E+08	Tubulin-specific chaperone D
**G%_drought^e^**	1	48.1	SCRI_RS_189898	358149096	HORVU1Hr1G048400	3.58E+08	3.58E+08	Methyltransferase-like protein 17, mitochondrial
**Reduction_G%**	1	48.1	SCRI_RS_189920	358151994	HORVU1Hr1G048410	3.58E+08	3.58E+08	Zinc finger HIT domain-containing protein 2
**Reduction_G%**	1	48.1	BOPA2_12_30694	375075126	HORVU1Hr1G050580	3.75E+08	3.75E+08	DnaJ homolog subfamily C member 13
**Reduction_G%**	1	48.1	BOPA1_4716–1205	375902959	HORVU1Hr1G050650	3.76E+08	3.76E+08	ARM repeat superfamily protein
**SL_DTI^f^ SL_drought^g^ Reduction_SL**	1	48.1	BOPA2_12_30406	376377160	HORVU1Hr1G050760	3.76E+08	3.76E+08	Inositol-tetrakisphosphate 1-kinase 1
**SL_DTI SL_drought Reduction_SL**	2	12.5	SCRI_RS_231806	18835372	HORVU2Hr1G009970	18833685	18835657	Eukaryotic aspartyl protease family protein
**SL_RL_control^h^ RL_DTI^i^**	2	12.7	SCRI_RS_108647	19617313	HORVU2Hr1G010380	19616032	19621632	FACT complex subunit SPT16
**RL_DTI**	2	12.7	SCRI_RS_155957	19671074	HORVU2Hr1G010400	19668641	19672131	Chromosome 3B, genomic scaffold, cultivar Chinese Spring
**SL_control^j^**	2	112.2	SCRI_RS_149429	710086215	HORVU2Hr1G023640	71041063	71041784	Chitinase family protein
**SL_control SL_control**	2	112.2	SCRI_RS_151556	710148626	HORVU2Hr1G023650	71043847	71046826	glycerol-3-phosphate acyltransferase 3
**SL_RL_drough^k^**	2	114.2	SCRI_RS_224454	713163055	HORVU2Hr1G023710	71400380	71406607	SNARE associated Golgi protein family
**GP_drought^l^ RL_drought Reduction_RL^m^**	2	114.9	SCRI_RS_223119	721191110	HORVU2Hr1G023840	72083404	72084531	rhomboid protein-related
**GP_control^n^**	2	118.7	BOPA2_12_31268	722327295	HORVU2Hr1G023850	72236010	72244224	BTB-POZ and MATH domain 2
**GP_drought**	2	119.8	SCRI_RS_55841	722894793	HORVU2Hr1G023890	72261989	72262321	Myosin-J heavy chain
**GP_control**	2	120	SCRI_RS_119513	721944874	HORVU2Hr1G023840	72083404	72084531	rhomboid protein-related
**Reduction_G%; G%_DTI**	5	44.1	BOPA1_ConsensusGBS0527-5	363522847	HORVU5Hr1G046890	3.64E+08	3.64E+08	saposin B domain-containing protein
**Reduction_G%; G%_DTI**	5	44.1	SCRI_RS_161107	365144333	HORVU5Hr1G047050	365071131	365072781	Plastid transcriptionally active 6
**Reduction_G%; G%_DTI**	5	44.2	SCRI_RS_223100	359525804	HORVU5Hr1G046260	359527202	359529307	Acetyltransferase NSI
**Reduction_G%; G%_DTI**	5	44.2	BOPA2_12_30193	368181264	HORVU5Hr1G047410	368180418	368183640	Protein phosphatase 2C family protein
**Reduction_G%; G%_DTI**	5	44.9	SCRI_RS_224213	369164317	HORVU5Hr1G047490	369158113	369161235	Zinc finger HIT domain-containing protein 1 homolog

^a^RL_drought—Root Length_drought

^b^Reduction_SL—Reduction_Shoot Length

^c^G%_DTI—Germination percentage_Drought Tolerance Index

^d^Reduction_G%—Reduction_ Germination percentage

^e^G%_drought—Germination percentage_drought

^f^SL_DTI—Shoot Length_ Drought Tolerance Index

^g^SL_drought—Shoot Length_ drought

^h^SL_RL_control—Shoot Length_ Root Length_control

^i^RL_DTI—Root Length_ Drought Tolerance Index

^j^SL_control—Shoot Length_control

^k^SL_RL_drought—Shoot Length_ Root Length_drought

^l^GP_drought—Germination Pace_drought

^m^Reduction_RL—Reduction_ Root Length

^n^GP_control—Germination Pace_control.

^o^Chr—Chromosome

^p^Pos—position in cM

^q^SNP Pos—Single Nicleotide Polymorphism position

#### Germination pace

**Fig 4:**
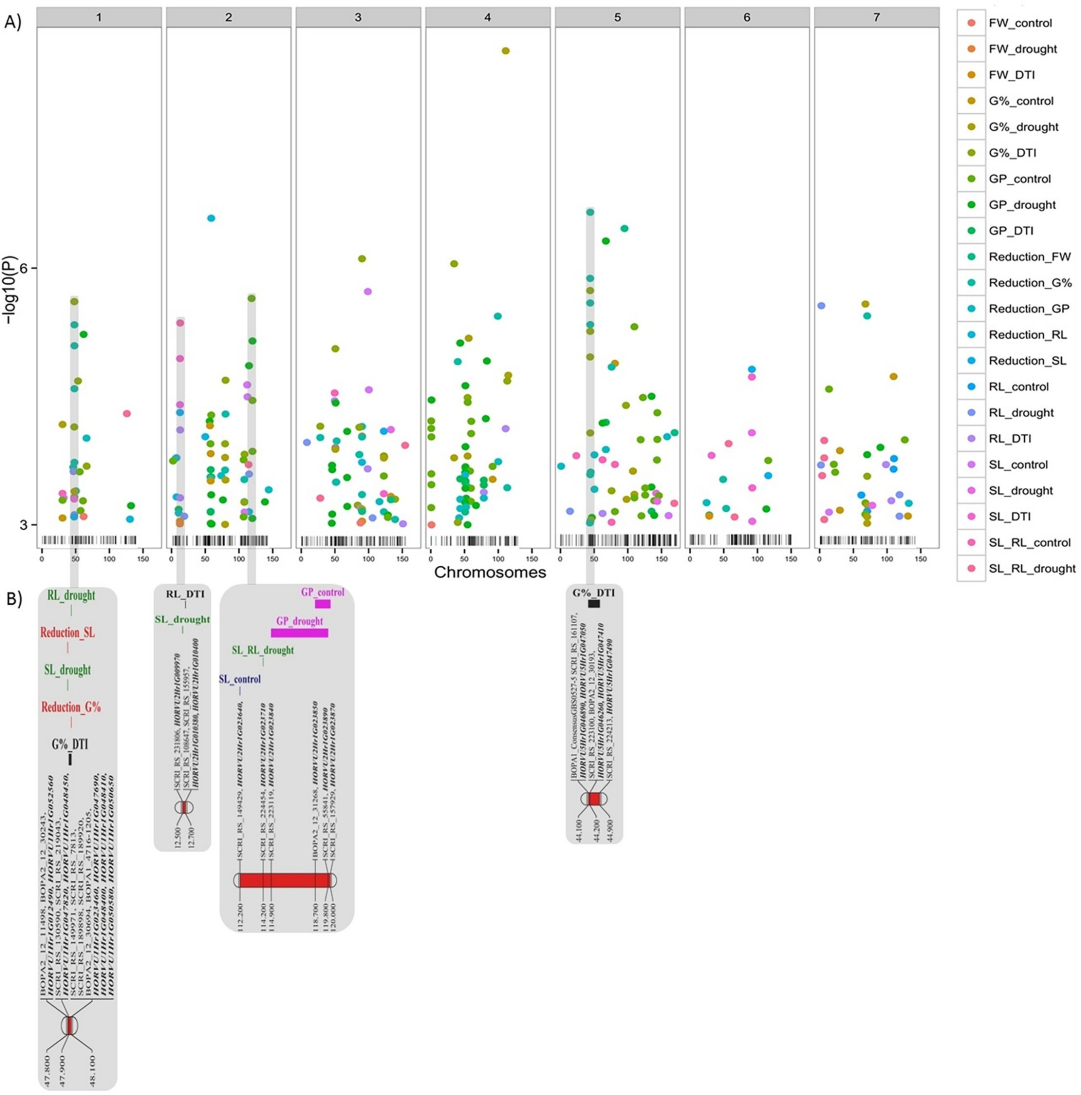
**A) The significant SNPs (338 SNPs, -log**_**10**_ **≥3) associated with all traits under control and drought stress conditions. The x-axis shows the chromosomes and the SNP positions. The y-axis shows the −Log10 (*P*-value) for each SNP marker. B) The candidate genes, which show a consistent effect on traits and associated with SNPs passing ≥ FDR using their physical position within 0.5 Mbp (~ 0.1 cM) were considered as highly putative candidate genes.** FW_control—Fresh Weight_control; FW_drought—Fresh Weight_drought; FW_DTI—Fresh Weight_Drought Tolerance Index; G%_control—Germination percentage_control; G%_drought—Germination percentage_drought; G%_ DTI—Germination percentage _ Drought Tolerance Index; GP_control—Germination Pace_control; GP_drought—Germination Pace _drought; GP_ DTI—Germination Pace_ Drought Tolerance Index; Reduction_FW—Reduction _ Fresh Weight; Reduction_ G%—Reduction _ Germination percentage; Reduction_ GP—Reduction _ Germination Pace; Reduction_RL—Reduction _ Root Length; Reduction_SL—Reduction _ Shoot Length; RL_control—Root Length_control; RL_drought—Root Length_drought; RL_ DTI—Root Length_ Drought Tolerance Index; SL_control—Shoot Length_control; SL_drought—Shoot Length_drought; SL_ DTI—Shoot Length_Drought Tolerance Index; SL_RL_control—Shoot Length_ Root Length_control; SL_RL_drought—Shoot Length_ Root Length_drought.

Totally, 135 SNPs were associated (–log_10_ p-value ≥3) with germination pace parameters (Panel A in S1 Fig). Fifty-two SNPs were detected on chromosomes 1 to 7 under control conditions. The highest effect was seen on chromosomes 2 and 5 at 118.7cM and 109.7cM (–log_10_ p-values of = 5.31, and 5.64, respectively), while 54 SNPs were identified on chromosomes 1 to 7 under drought stress conditions. The most significant effect (with–log_10_ p-value = 6.31) was observed on chromosome 5 at 67.4cM. For GP_DTI, nine SNPs were identified on chromosomes 2, 4 and 5, while the most significant one (with–log_10_ p-value = 4.19) was observed on chromosome 5 at 67.4cM. For Reduction_ GP, twenty SNPs were detected spread across all seven chromosomes (Fig 4). One gene was control-specific, namely *HORVU2Hr1G023850* and one was drought specific *namely HORVU2Hr1G023890*. *HORVU2Hr1G023840* was identified as a constitutive gene for GP_control and under drought (Table 3 and S3 Table).

#### Comparisons between genotypes according to their geographical origin, biological status and row-type

Based on geographical origin, the high values were detected at Europe for G% and RL, EA for SL, and AM for FW. For biological status, the larger values were detected at breeding material for all seedling traits except G% where slightly similar values were found in breeding material and improved cultivar. Ultimately, based on row-type, the high values were detected at six-row for SL and FW, two-row for G%, and slightly similar values for two and six-rows were found in RL (Panel A, B & C in S2 Fig).

### Shoot length

GWAS found 29 SNPs associated with shoot length parameters. Eight SNPs were mapped on chromosomes 2, 3, 5 and 7 SNPs for SL under control conditions, where the most significant one (with–log10 p-value = 5.72) was observed on chromosome 3 at 98.9cM (Panel B in S1 Fig). Under drought stress, 10 SNPs were observed mapping on chromosomes 1, 2, 3, 5, 6 and 7. For SL_DTI, six SNPs were detected on chromosomes 2, 3, and 6. The most significant SNPs (with–log10 p-value = 4.72 and 4.94, respectively) are located on chromosomes 2 and 6 at 12.5cM and 91.7cM, respectively (Fig 4). Two constitutive genes, namely *HORVU1Hr1G050760* for SL_DTI and *HORVU1Hr1G052560* for Reduction_SL, one drought-specific, namely (*HORVU1Hr1G050760*) for SL_drought, and three control-specific, namely (*HORVU2Hr1G023640* and *HORVU2Hr1G023650*) for SL_control were associated (Table 3 and S3 Table).

### Root length

Thirty-six SNPs were associated (p-value ≤ 0.001) with root length parameters (Panel B in S1 Fig). Under control conditions, five SNPs were mapped on chromosome 7 for RL,while 11 SNPs were mapped on chromosomes 1, 2, 3, 5 and 7 for drought-induced stress. Here, the most significant SNP (with–log_10_ p-value = 5.56) is positioned on chromosome 7 at 2.5cM. For RL_DTI, 12 SNPs were mapped for RL-DTI on chromosomes 2, 3, 4, 5,and 7. Three genes are constitutive genes, i.e. *HORVU2Hr1G010380* and *HORVU2Hr1G010400* for RL_DTI,*HORVU2Hr1G023840* for reduction of RL, and three are drought-specific, i.e. *HORVU1Hr1G012490*, *HORVU1Hr1G023460*, and *HORVU2Hr1G023840* for RL_drought. No genes were identified for RL under control (Table 3 and S3 Table).

#### Shoot-root ratio

For shoot-root ratio parameters, 19 SNPs showed a significant association with–log_10_ p-value ≥3 (Panel B in S1 Fig). Under control conditions, 8 SNPs were detected on chromosomes 1, 2, 3 and 5. The two most significant effects (with–log_10_ p-value = 4.54, and 5.35, respectively) were found on chromosomes 2 and 3 at 12.7cM and 49.7cM, respectively, while 11 SNPs were mapped on chromosomes 1, 2, 3, 5, 6 and 7 under drought-induced stress (Fig 4). Only two SNPs showed an association with candidate genes: one was control-specific, (*HORVU2Hr1G010380*), and the other drought-specific; (*HORVU2Hr1G023710*) for SL_RL ratio (Table 3 and S3 Table).

### Fresh weight

In total, 15 SNPs were associated with fresh weight parameters (Panel C in S1 Fig). Under control conditions, 3 SNPs were detected on chromosomes 1, 3 and 4 for FW. Under induced drought, only two SNPs were identified on chromosomes 2 at 12.5cM and 3 at 90.3cM. Four SNPs were mapped for FW_DTI on chromosomes 2, 3 and 6. For Reduction_FW, six SNPs were detected, the most significant one (with–log_10_ p-value = 6.46) being observed on chromosome 5 at 95.1cM (Fig 4). There are no SNPs associated with candidate genes for fresh weight parameters.

### Candidate genes

Through a combination of genetic and physical maps, it was possible to locate the position significant SNP markers in the barley reference sequence. In this way, we were able to find twenty-six regions for genetic variation of the traits studied that harbor candidate genes. Based on these findings, 9 adaptive genes are control-specific or drought-specific. We could also find 17 constitutive genes involved in the genetic variation of the traits studied under both control and induced drought. On chromosome 1, no control-specific genes were identified, while four of them are drought-specific and the remaining 9 are constitutive. On chromosome 2, the genes are a combination of both categories: 8 adaptive and 3 constitutive genes. On chromosome 5, all of the genes are constitutive (Table 3 and S3 Table). Putative candidate genes in this study, especially those that are drought-specific, are very promising for a wider application in crop molecular breeding.

## Discussion

The genotypes used in the current study show a wide range of phenotypic variation at the early developmental stage in response to drought-induced stress. The population we used is known as a highly diverse collection, which has repeatedly been employed for GWAS to detect QTL for adaptive and developmental traits e.g. leaf blade area [25]. Induced drought had a considerable impact on most of the traits, especially in G% and GP compared to controls, most likely PEG-induced drought decreases water uptake and consequently germination, which becomes either delayed or occurs at a reduced rate [34,35]. Under induced drought conditions, a high percentage and rate of germination in barley are essential for vigorous stand development in the early stages [4].

Moreover, in the current study, drought clearly has a negative impact on seedling performance by inhibiting shoot and root-related development. A wide genetic variation has been reported in the seminal roots of wild and cultivated barley under induced stress conditions [36]. In cereals, plant growth performance was found to be positively associated with well-developed root systems, as well as early seedling vigor [11,12], both of which can help to improve stress tolerance. Thus, focusing on such traits would be an efficient approach for evaluating in a short time a large number of genotypes under drought conditions. Such a selection based on the shoot and root traits would be effective to identify genotypes for a better performance under drought stress conditions.

Functional validation of candidate associations, e.g., re-sequencing the candidate genes or expression analyses will help to increase our knowledge about drought tolerance during germination. The genetic variation and high heritability of the shoot- and root-related traits found in the collection used here is an important resource for further molecular breeding work aimed at selecting drought tolerance accessions. These, in turn, could help in the improvement of barley performance under drought stress.

The comparisons between genotypes according to geographic origin showed that the genes for drought tolerance are distributed among the genotypes that originate from EU, EA and AM. Based on the biological status, the breeding materials and advanced cultivars are more adapted to drought than the landraces. The six-rowed genotypes revealed better performance than the two-rowed ones. Overall, these findings indicate that there is no ideal genotype that compresses all desired traits. This can be achieved by crossing genotypes from different geographical origin to pyramid all desired traits in an elite genotype.

### Candidate genes

Based upon the latest barley reference genome sequence [33], several candidate genes were identified based on their physical position. The functional annotation of these genes in barley and orthologs in Arabidopsis confirm the role of these candidates with regard to drought tolerance at the germination and early seedling development stage (S3 Table). We will focus on the genes regulating seed germination and early growth in barley under drought stress. On chromosome 1, the most promising regions are located at position 46.6–48.1cM. Notably, there is a plenty of genetic variation in this region, which is important for many traits related to drought tolerance. This region harbors several candidate genes, such as *HORVU1Hr1G012490* for RL_drought, which encodes E3 ubiquitin-protein ligase SINA-like 11, and is known to be involved in drought tolerance as a positive regulator of ABA signaling in Arabidopsis and rice [37].

Moreover, three genes were identified as candidates for G%_DTI in this genomic region. The first one is *HORVU1Hr1G047820* encoding aldo-keto reductase family 4 member C9, AKRs Family, which has shown to be involved in abiotic stress-related reactive aldehyde detoxification pathways and is used for the improvement of stress tolerance in plants [38]. The second candidate *HORVU1Hr1G048450* encodes actin depolymerizing Factor 6. The expression of actin genes confers drought-stress tolerance to transgenic Arabidopsis seedlings by increasing their germination rate, primary root length, and survival [39]. In maize, Lü et al. [40] suggest that ADF could stimulate ABA biosynthesis by increasing osmotic stress tolerance since ADF plays a key role in cytoskeleton assembly during cell division [41]. It could be suggested that the decline in germination percentage is a consequence of a reduction in cell growth due to a decrease in the cytoskeleton and cell division-related proteins under drought stress.

The third gene, *HORVU1Hr1G048400*, encodes methyltransferase-like protein 17 (mitochondrial), which is known to be drought-responsive proteins involved in reactive oxygen species (ROS) scavenging, cell structure, and cycle, and increased response to drought tolerance [41]. *HORVU1Hr1G050760* encoding Inositol-tetrakisphosphate 1-kinase 1 belongs to the ITPK proteins that have been identified as the intermediate precursor of phytic acid PA biosynthesis [42,43]. During germination, PA is catabolized by phytase enzymes, allowing the remobilization of the sequestered minerals to support juvenile seedling growth [44]. The reduction of germination under drought might result from the inability of ITPK to release the seed-stored calcium that plays a pivotal role in the formation of new cell walls during mitotic division in plant response to drought [45]. Taking these results together, we can conclude that there is interplay between the ADF and ITPK. Whereas ADF induces the ABA biosynthesis under drought, the excess ABA accelerates the PA biosynthesis that in turn acts as an anti-nutritional agent by immobilizing the nutritional ions that inhibit seed germination.

Another very important and consistent trait-specific group of the gene for Reduction_G% was found on chromosome 1 at 48.1 cM. *HORVU1Hr1G048410* encodes zinc finger HIT domain-containing protein 2; the orthologs of this gene in Arabidopsis *AT1G03790* is a seed-specific gene that is an ABA-regulated gene that inhibits seed germination due to the accumulation of ABA [46]. Also, *HORVU1Hr1G050580* encodes DnaJ homolog subfamily C member 13, which has been identified as belonging to the heat shock proteins (Hsps). Kaur et al. [47] found that expression of rice heat shock protein OsHSP18.2 ensured high germination rates under drought in Arabidopsis by stabilizing proteins and overriding the deleterious effects of ROS. A set of the HsPs is differentially regulated under drought stress during different developmental stages in barley, indicating the pivotal role of chaperones for drought tolerance and as well as for development [48]. These findings imply that HsPs play an important role in seed germination in barley. The last gene for Reduction_G% in this genomic region, i.e. *HORVU1Hr1G050650*, encodes ARM repeat superfamily protein, which is important in signaling pathways under different abiotic stresses [49]. The CaPUB1 orthologs in Arabidopsis reveal similar interaction under both drought and salinity, suggesting that this ARM/E3 mediates different abiotic stresses [50,51].

We can conclude that the putative candidate genes on chromosome 1 can be classified into two categories: germination-specific genes and post-germination genes. The first category regulates the variation of the germination related-parameters, especially, the reduction in G% from 90% under control to 74% under drought treatment (S1 Table), while the second category modulates the variation of seedling traits, particularly root length. This conclusion is supported by very low correlation between RL_drought and G%_drought r = 0.02 (Fig 3b). Together, these genes encode different proteins families and regulate different defense mechanisms to maintain seed germination, including ROS scavenging, signaling, and ubiquitination. More likely, there is crosstalk and synergism between these mechanisms that work to mitigate the consequences of drought.

On chromosome 2, the candidate gene is *HORVU2Hr1G009970* encoding eukaryotic aspartyl protease family protein for SL_DTI, SL_drought, and Reduction_SL. Yao et al. [52] report that APs may function in drought avoidance through ABA signaling and several proteases increase under drought stress, such as ATP-dependent Clp protease in *H*. *vulgare*, cysteine proteinase in *P*. *vulgaris*, zinc metalloprotease in *B*. *napus*, and aspartic proteinase in *Z*. *mays*. It is likely that this gene is shoot-specific and drought-specific because it controls the variation of shoot parameters under drought, exclusively. This finding suggests that this gene can be employed to improve shoot related-traits in barley to be grown under drought. The second candidate gene on chromosome 2 was *HORVU2Hr1G010400*, which encodes Chromosome 3B, genomic scaffold, cultivar Chinese Spring, which shows an association with RL_DTI. The orthologous gene in Arabidopsis AT2G36270 "ABA insensitive 5" is a basic-lucine transcription factor that is responding to water deprivation and salt stress. ABI5 acts as a negative regulator of seed germination and post-germination growth retardation [53].

*HORVU2Hr1G023710* encodes SNARE-associated Golgi protein family, soluble N-ethylmaleimide-sensitive factor (NSF) attachment protein receptors, which is a candidate for SL_RL_drought. This protein family is known as a vesicle transport family protein involved in cell homeostasis under osmotic stress in rice [54]. This vesicle traffic plays a key role in cell homeostasis, growth,and the development of plants [55]. Similarly, the bet-like SNARE- AtBS14a has been found to be significant in controlling cell growth in Arabidopsis [56]. The overexpression of SNARE-like protein from the halophyte *Salicornia brachitata* conferred salinity/drought tolerance on transgenic tobacco lines by enhancing seed germination, membrane stability, antioxidant enzymes coding genes expression,and maintaining cell turgor [57].

Interestingly, *HORVU2Hr1G023840* encodes a rhomboid protein-related, gene, revealing a pleiotropic genetic control and constitutive pattern, as it controls the variation of several traits, and mediates a trait variation under control and drought (Table 3). This result is consistent with the findings published by Soda et al. [58] who found that *OsRhmbd2*-*putative Rhomboid homolog* (*LOC_Os01g16330*) showed a high constitutive expression in Pokkali under control and salinity stress. In rice, Wang et al. [59] found that rhomboid proteases were induced in response to drought in both leaves and roots at all developmental stages. These findings indicate that this gene is of high importance because it can be used to enhance barley development by improving the germination potential and seedling development under stressed and non-stressed conditions. Another gene controlling GP_droguht is *HORVU2Hr1G023890*, which encodes myosin-J heavy chain. The abundance of myosin heavy chain-related proteins significantly increased in the Diamond plants under cold stress [60]. In rice seedlings, Yan [61] observed an increase in the intensity of myosin-like proteins under chilling stress. Probably, this gene responds to different cues of abiotic stresses.

These results suggest that, the genes on chromosome 2 are orchestrating the variation in shoot and root parameters rather than the variation in germination related parameters (Table 3). This is supported by the high correlation between SL-drought with SL_RL ratio r = 0.66 and the significant correlation with RL_drought r = 0.49* (Fig 3B).

On chromosome 5, five genes were identified as controlling the variation of G%_DTI and Reduction_G% (Table 3). All of these genes are constitutive and germination-specific; we could identify the functions of two genes. The first gene *HORVU5Hr1G046890* encodes saposin B domain-containing protein. In sugarcane, three genes encoding saposin B domain-containing proteins exhibited overexpression under water deficit and all of them are involved in the lipid metabolism [62]. Saposin-like type B region 1 family protein is thought to protect cells against drought stress by altering the lipid composition of the plasma membrane and stress-induced fatty acid unsaturation [63]. The second gene *HORVU5Hr1G047050* encodes plastid transcriptionally active 6. Shortly after imbibition, the plastid transcriptional machinery is switched on and three RNA polymerases begin the transcription of photosynthesis-related genes [64]. During germination, the accumulated mRNAs are not followed by translation into proteins, indicating that the excess mRNAs are more helpful for germination than it is for proteins [65].

All genes on chromosome 5 control the variation of G% related parameters; Reduction_G% and G%_DTI (Table 3). Probably, this category of genes accounting for the large portion of reduction in G% from 90% under control to 74% under drought (S1 Table).

Overall, barley has developed different mechanisms to ameliorate the deleterious impact of drought during seed germination and early growth. Our findings show that this panel of genes can be harnessed to improve the drought tolerance of barley. The candidate genes on chromosome 1 and 5 can be used to improve the germination percentage, especially those of chromosome 5 because they control the variation in G% exclusively. To improve the germination pace, the candidates on chromosome 2 are relevant. The seedling-related traits,such as shoot/root parameters can be improved by employing certain genes on chromosomes 1 and 2 only.

## Conclusion

The present study shows how drought stress can affect seed germination and seedling parameters during early growth. The measured parameters showed high heritability values, suggesting that they would be used as selection parameters to test a large number of genotypes in a short time. The genetic analyses of seed germination under drought stress emphasize how these traits are genetically complex. We identified drought stress-responsive genes encoding different proteins that regulate the germination and post-germination events. The identified genes modulate seed germination under control and induced drought in two different ways, namely constitutively or adaptively. The constitutive genes can be harnessed for selection, either under control or drought, especially those on chromosome 5. Pinpointing the regulated genes of adaptation is thought to be essential for drought stress responses at an early stage in plant development and it can be used in genetic manipulation to improve barley plant tolerance. Based on the comparison between the genotypes according to their geographical origin, biological status and row type, the six rows breeding materials and advanced cultivars originated from EU, EA and AM can be used to improve germination percentage, root length and shoot length, respectively. These findings demonstrate that barley seedlings use sophisticated mechanisms for adaptation at an early developmental stage. Further functional validation analysis to confirm these candidate associations/genes found in this work is required to understand the genetic control of drought tolerance at early developmental stages (germination and seedling) in cereals. Notwithstanding, field trials will be essential to critically validate our results in terms of their agronomic importance.

## Supporting information

S1 Table Phenotypic information for 218 spring world-wide barley collection. (XLSX)

S2 Table The list of significant SNPs associated with all traits under control and drought stress conditions. (XLSX)

S3 Table The detailed information about the functional annotation of the putative candidate genes in barley and Arabidopsis associated with the studied traits under drought and control growth conditions. (XLSX)

S1 Fig A: Manhattan plot of (A1) G%_control—Germination percentage_control, (A2) G%_drought—Germination percentage_drought, (A3) G%_DTI—Germination percentage_Drought Tolerance Index, (A4) Reduction_G%—Reduction_ Germination percentage, (A5) GP_control—Germination Pace_control, (A6) GP_drought—Germination Pace_drought, (A7) GP_ DTI—Germination Pace_ Drought Tolerance Index and (A8) Reduction _GP—Reduction_Germination Pace traits evaluated under control and drought conditions. B: Manhattan plot of (B1) SL_control—Shoot Length_control, (B2) SL_drought—Shoot Length_drought, SL_DTI—Shoot Length_Drought Tolerance Index, (B3) Reduction_SL—Reduction_Shoot Length, (B4) RL_control—Root Length_control (B5) RL_drought—Root Length_drought, (B6) RL_DTI—Root Length_Drought Tolerance Index, (B7) Reduction_RL—Reduction_Root Length, (B8) SL_RL_control—Shoot Length/Root Length_control, (B9) and (B10) SL_RL_drought—Shoot Length/Root Length_drought traits, evaluated under well-watered and stress-watered conditions. C: Manhattan plot of (C1) FW_control—Fresh Weight_control, (C2) FW_drought—Fresh Weight_ drought, (C3) FW_DTI—Fresh Weight_ Drought Tolerance Index and (C4) Reduction_FW—Reduction_Fresh Weight traits, under control and drought conditions. The x axis shows the chromosomes and the SNP order. The y-axis shows the −Log10 (P-value) for each SNP marker.
(PDF)

S2 Fig A: Boxplot analysis of variation of the traits based on geographical origin; A1) Germination percentage_DTI, A2) Root Length_ DTI, A3) Shoot Length_ DTI, A4) Fresh Weight_ DTI in barley genotypes. B: Boxplot analysis of variation of the traits based on biological status; B1) Germination percentage_DTI, B2) Root Length_ DTI, B3) Shoot Length_ DTI, B4) Fresh Weight_ DTI in barley genotypes. C: Boxplot analysis of variation of the traits based on row-type; C1) Germination percentage_DTI, C2) Root Length_ DTI, C3) Shoot Length_ DTI, C4) Fresh Weight_ DTI in barley genotypes.
(PDF)
