# Supplementary figures and images for: Genetic basis of drought tolerance during seed germination in barley
Source: PLoS One. 2018 Nov 2;13(11):e0206682. doi: 10.1371/journal.pone.0206682 (PMC6214555; doi:10.1371/journal.pone.0206682)

S1A Fig.

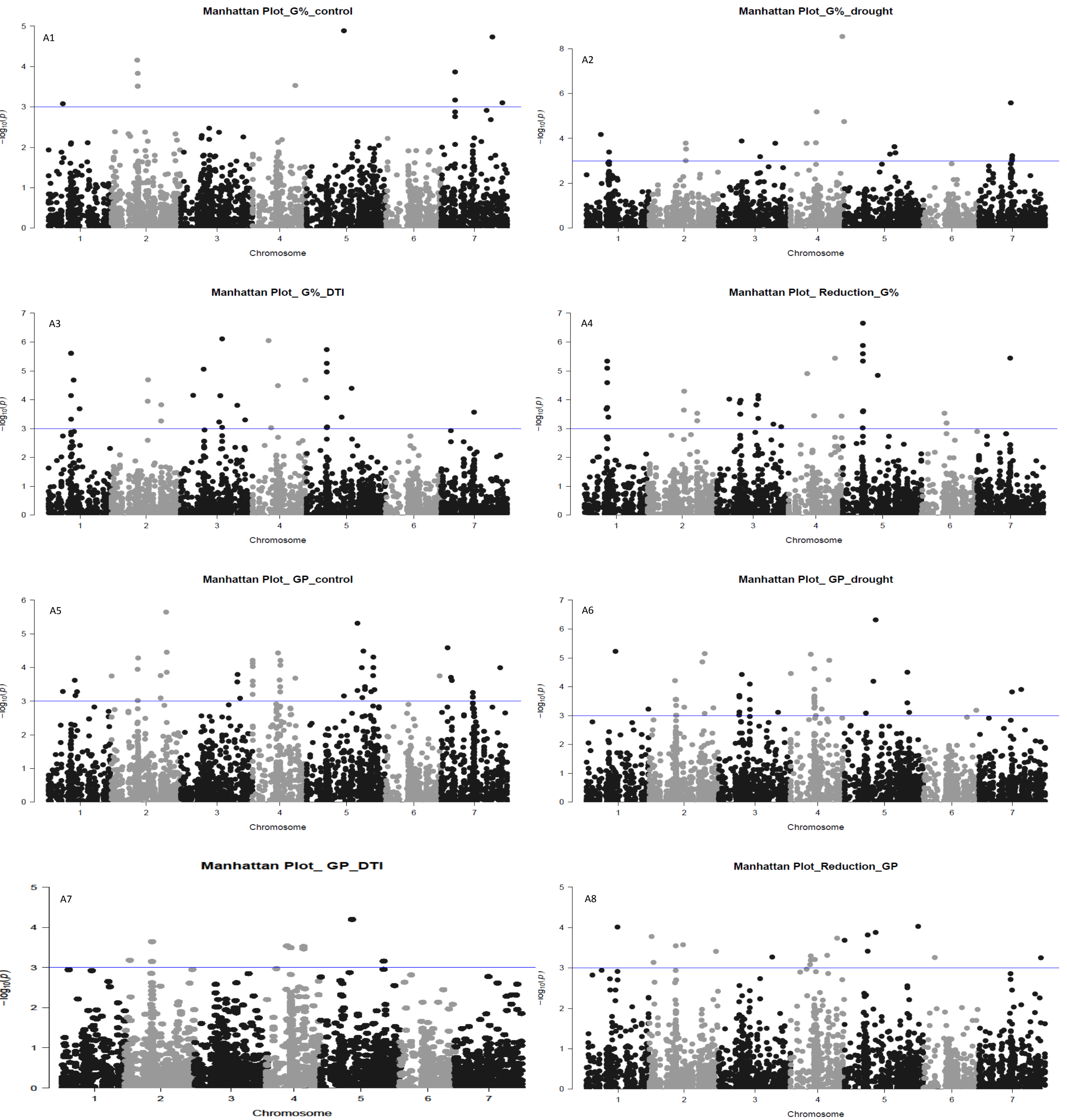

S1B Fig.

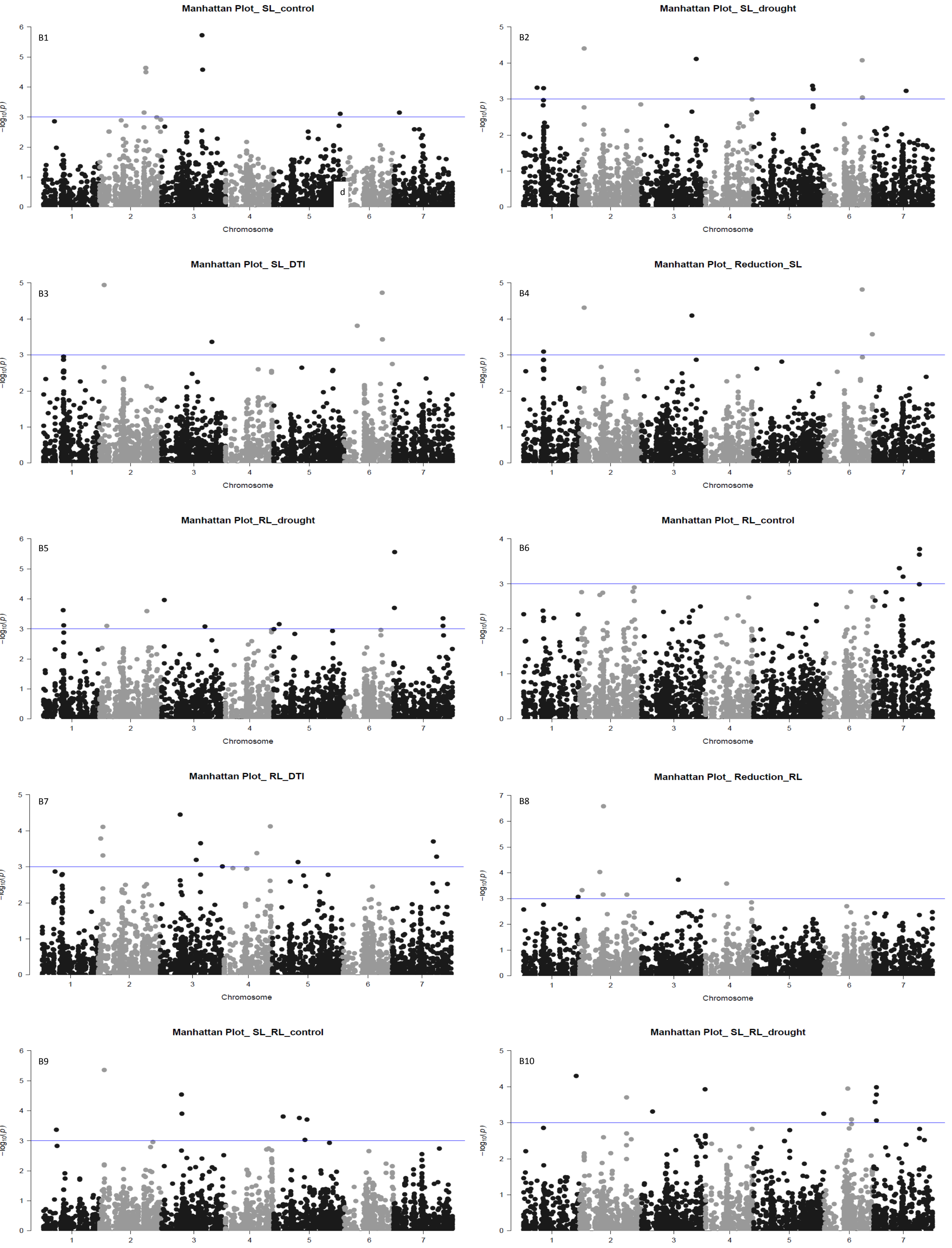

S1C Fig.

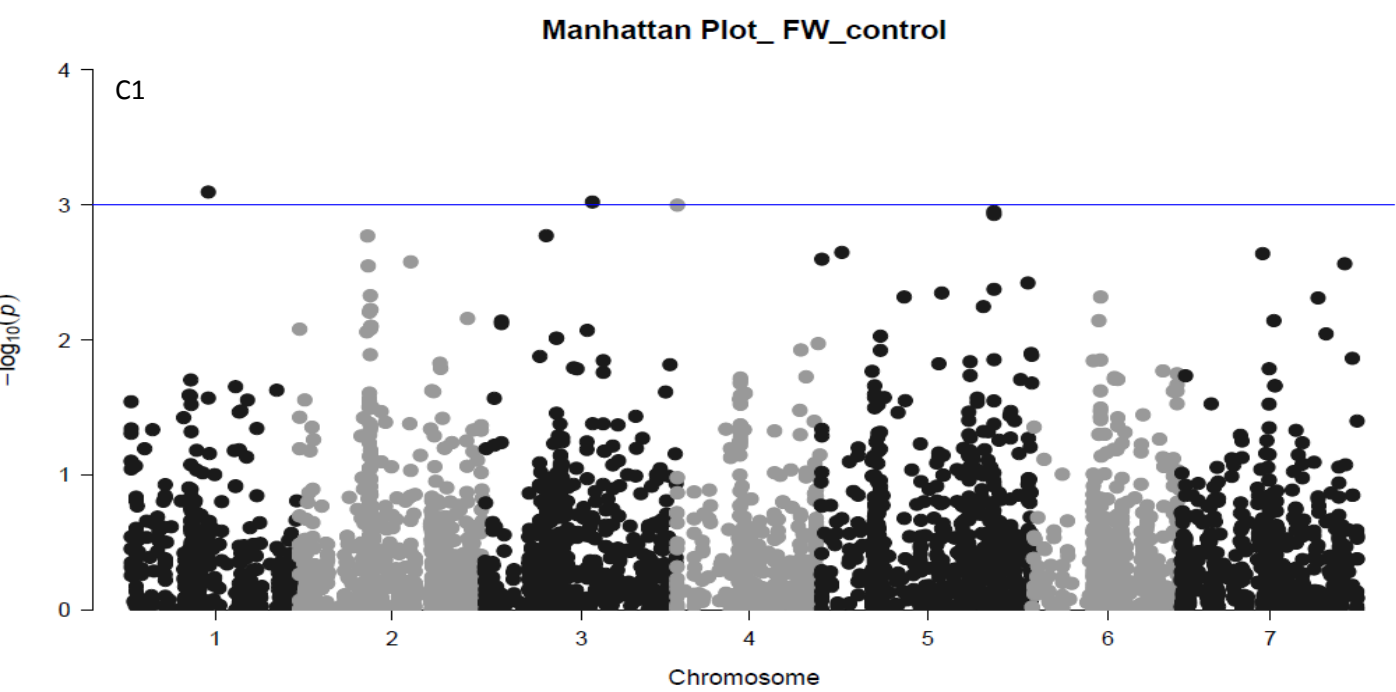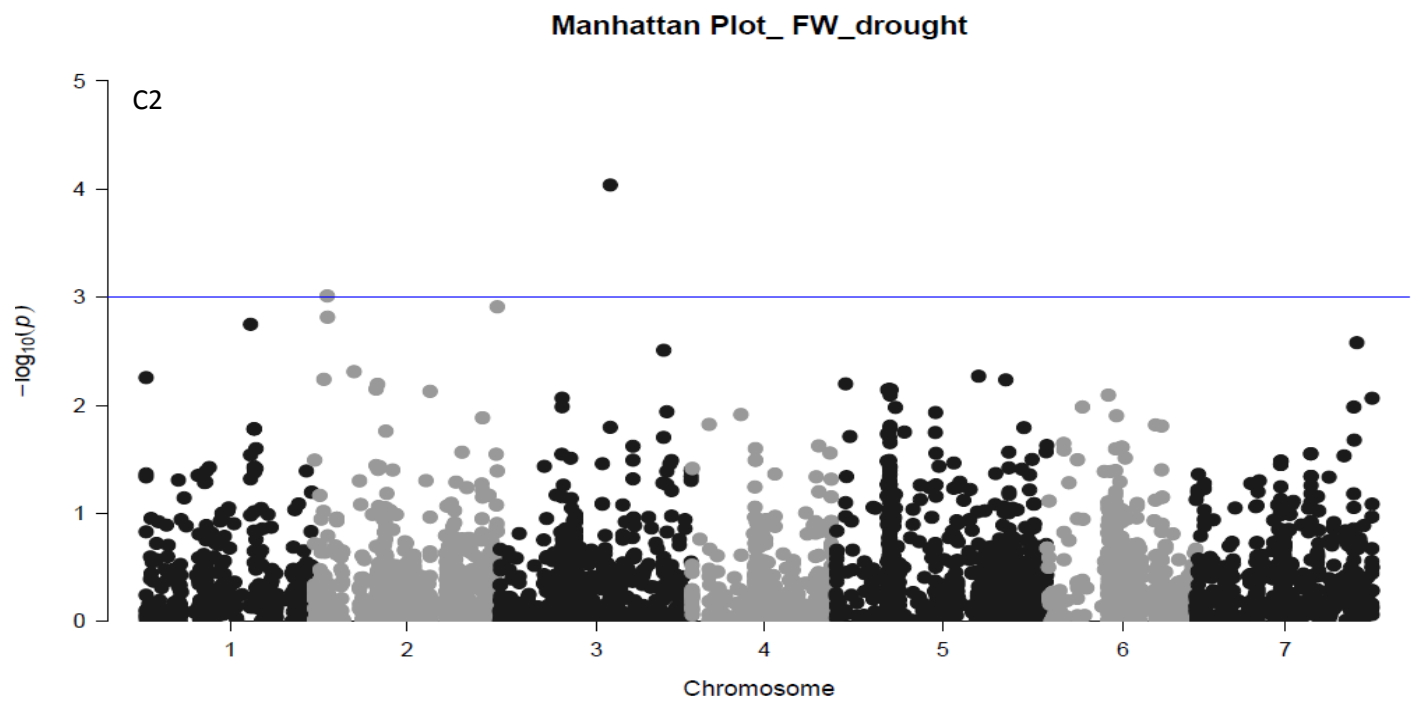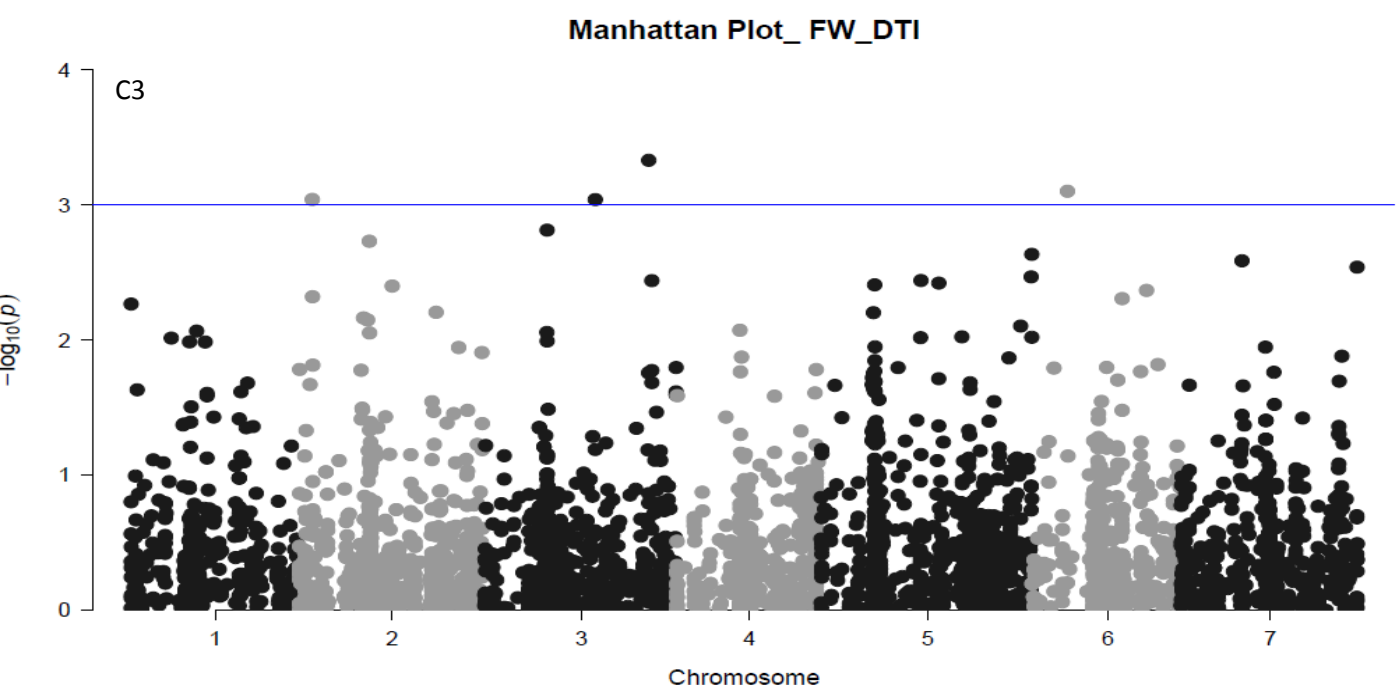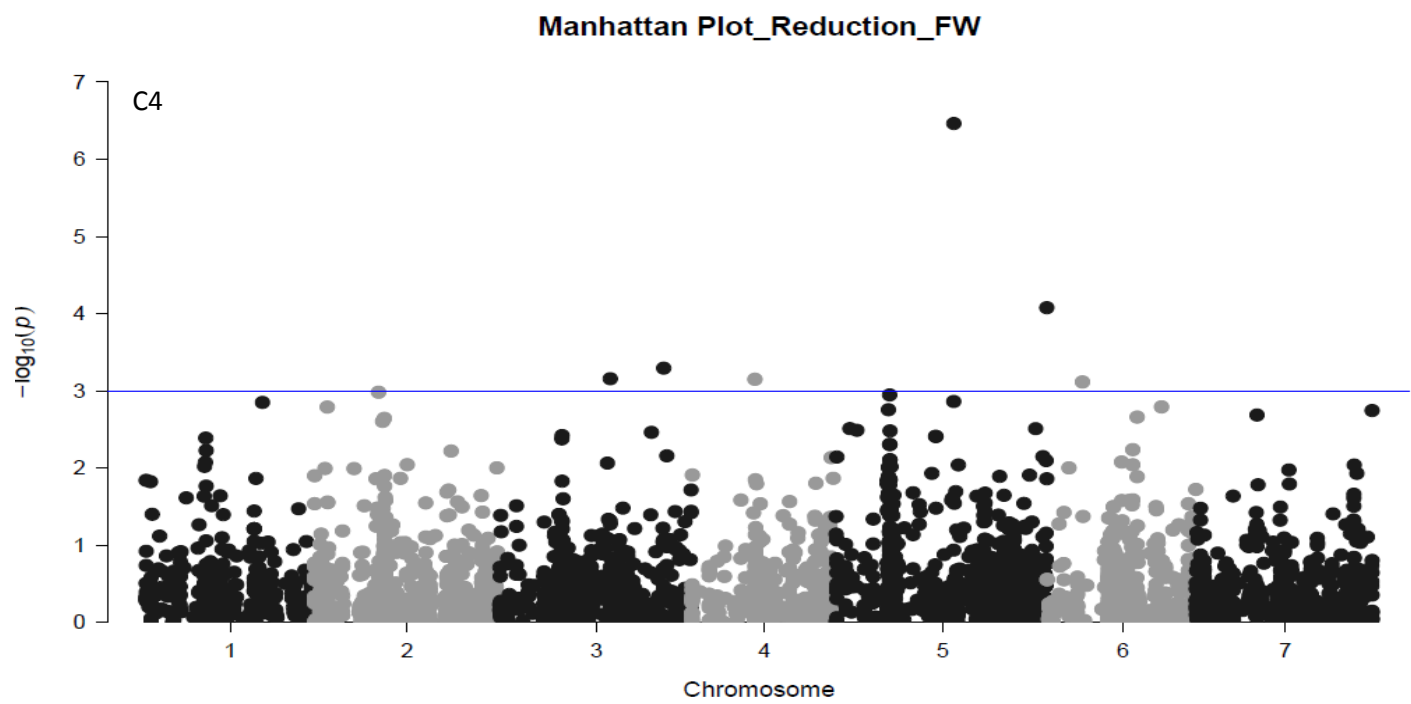

Supplement: S1 Fig — A: Manhattan plot of (A1) G%_control—Germination percentage_control, (A2) G%_drought—Germination percentage_drought, (A3) G%_DTI—Germination percentage_Drought Tolerance Index, (A4) Reduction_G%—Reduction_ Germination percentage, (A5) GP_control—Germination Pace_control, (A6) GP_drought—Germination Pace_drought, (A7) GP_ DTI—Germination Pace_ Drought Tolerance Index and (A8) Reduction _GP—Reduction_Germination Pace traits evaluated under control and drought conditions. B: Manhattan plot of (B1) SL_control—Shoot Length_control, (B2) SL_drought—Shoot Length_drought, SL_DTI—Shoot Length_Drought Tolerance Index, (B3) Reduction_SL—Reduction_Shoot Length, (B4) RL_control—Root Length_control (B5) RL_drought—Root Length_drought, (B6) RL_DTI—Root Length_Drought Tolerance Index, (B7) Reduction_RL—Reduction_Root Length, (B8) SL_RL_control—Shoot Length/Root Length_control, (B9) and (B10) SL_RL_drought—Shoot Length/Root Length_drought traits, evaluated under well-watered and stress-watered conditions. C: Manhattan plot of (C1) FW_control—Fresh Weight_control, (C2) FW_drought—Fresh Weight_ drought, (C3) FW_DTI—Fresh Weight_ Drought Tolerance Index and (C4) Reduction_FW—Reduction_Fresh Weight traits, under control and drought conditions. The x axis shows the chromosomes and the SNP order. The y-axis shows the −Log10 (P-value) for each SNP marker. (PDF) [file pone.0206682.s004.pdf]

S2A Figs

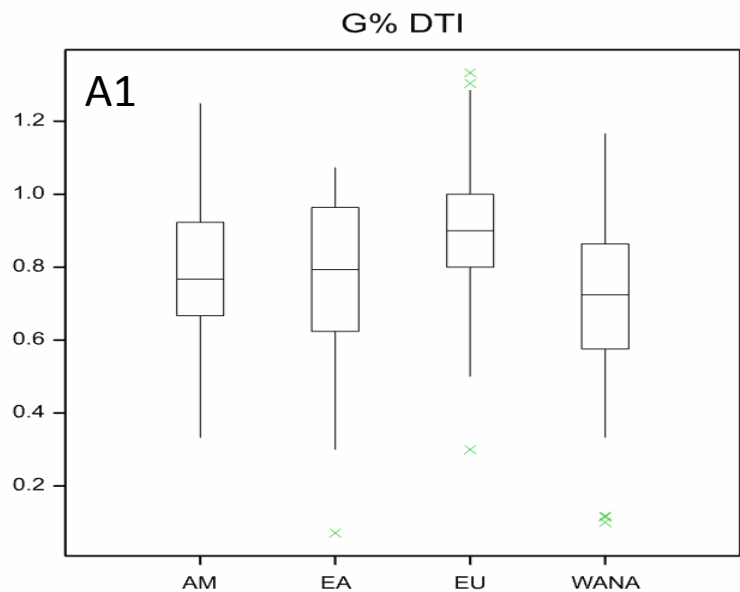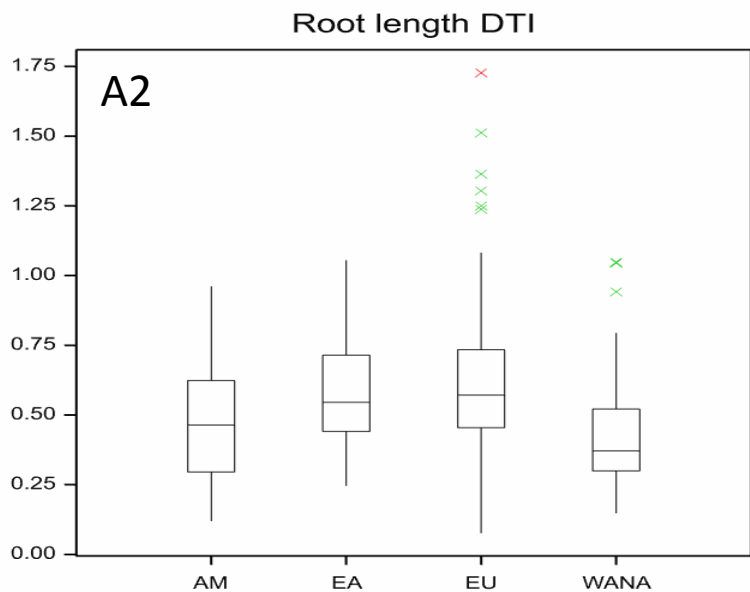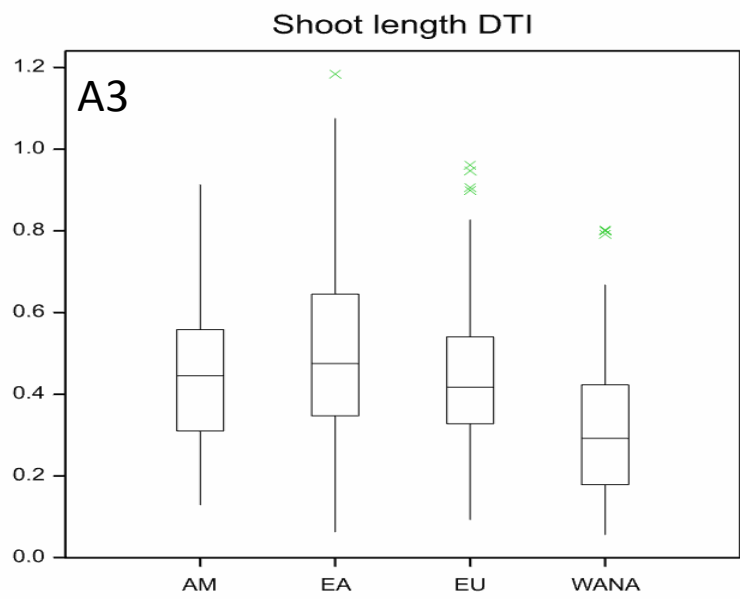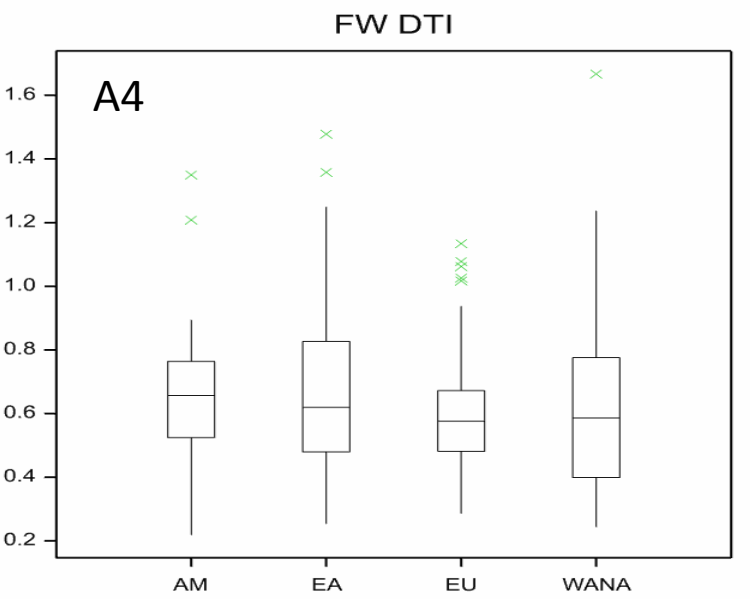

S2B Figs

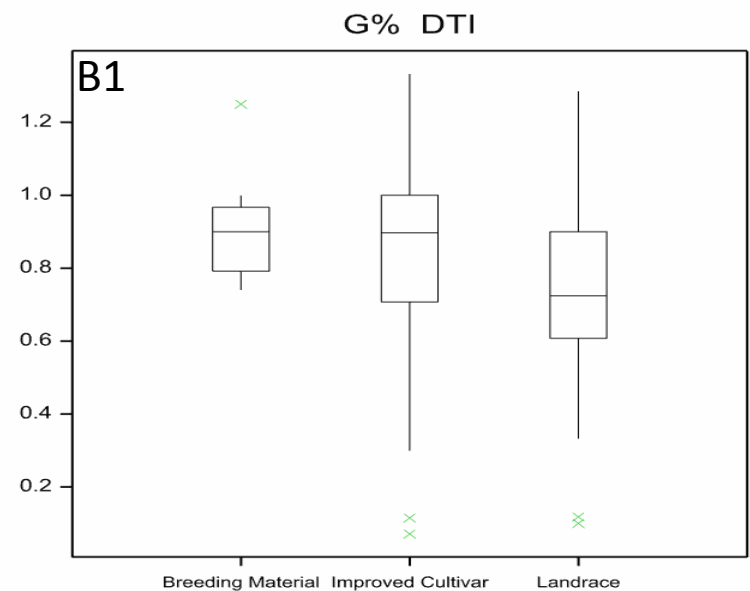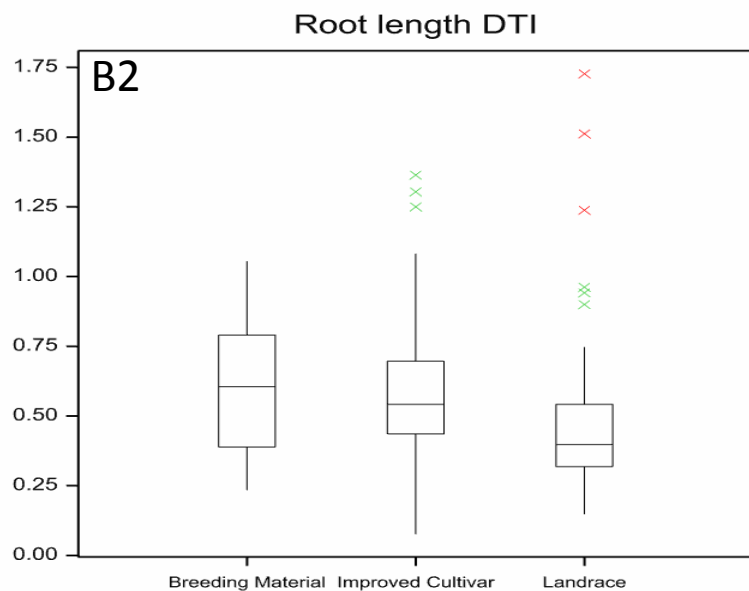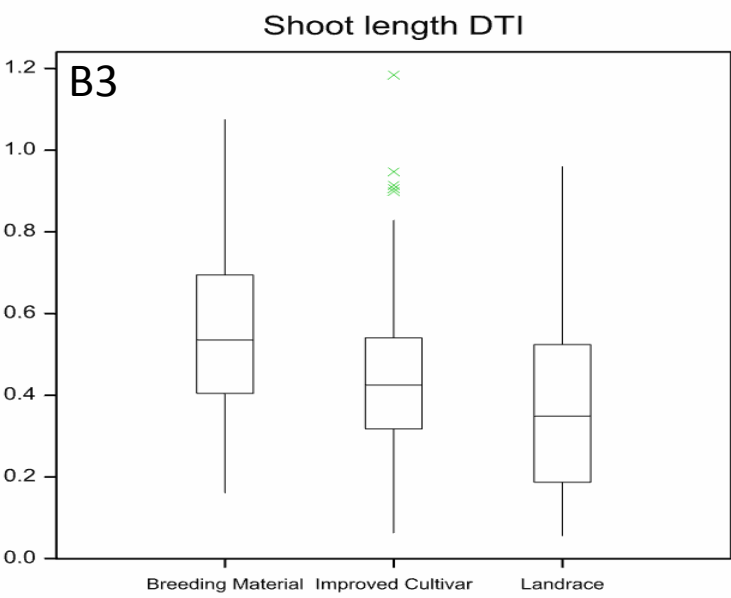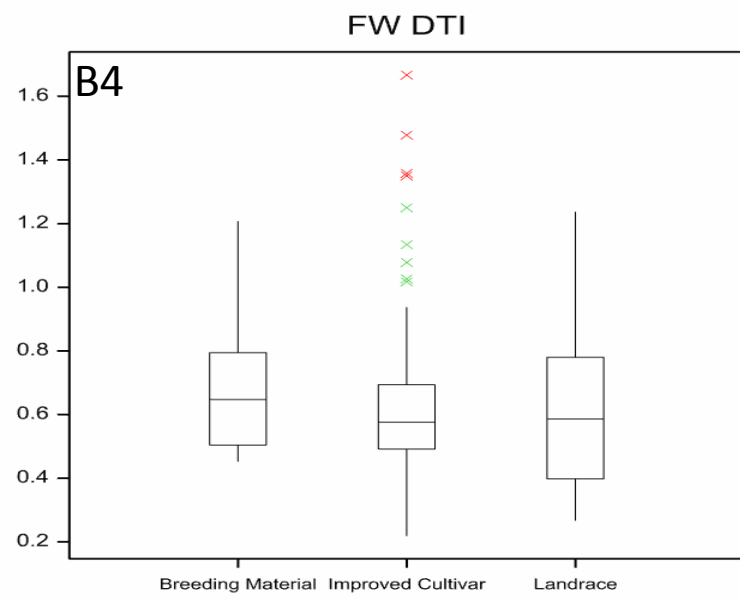

# S2C Figs

G% DTI

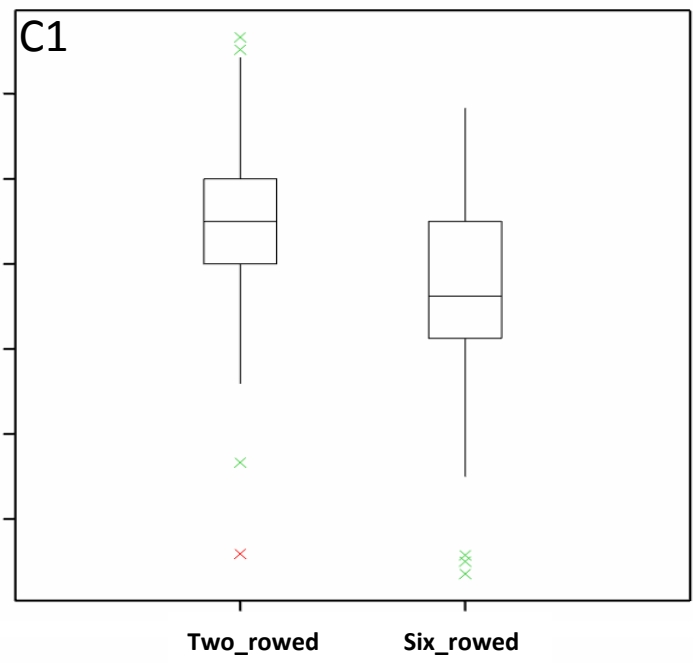

Root length DTI

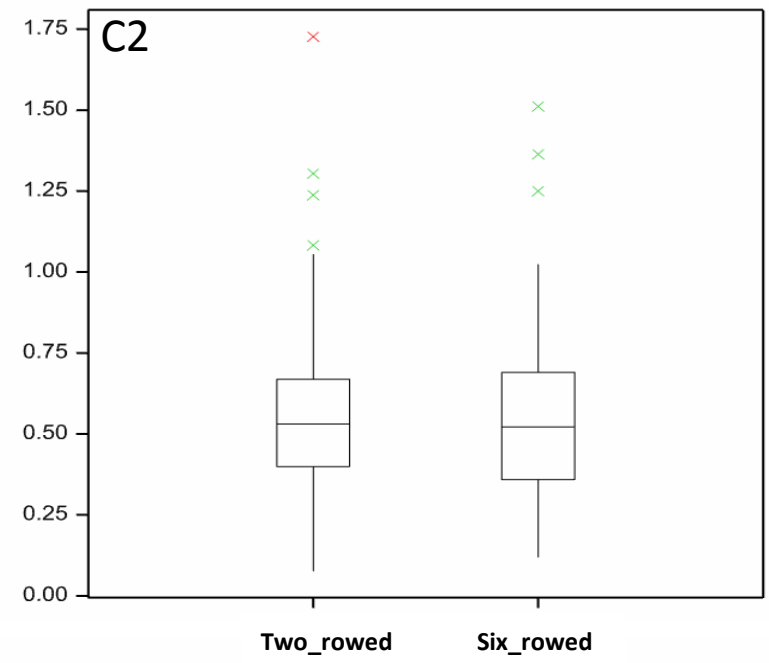

Shoot length DTI

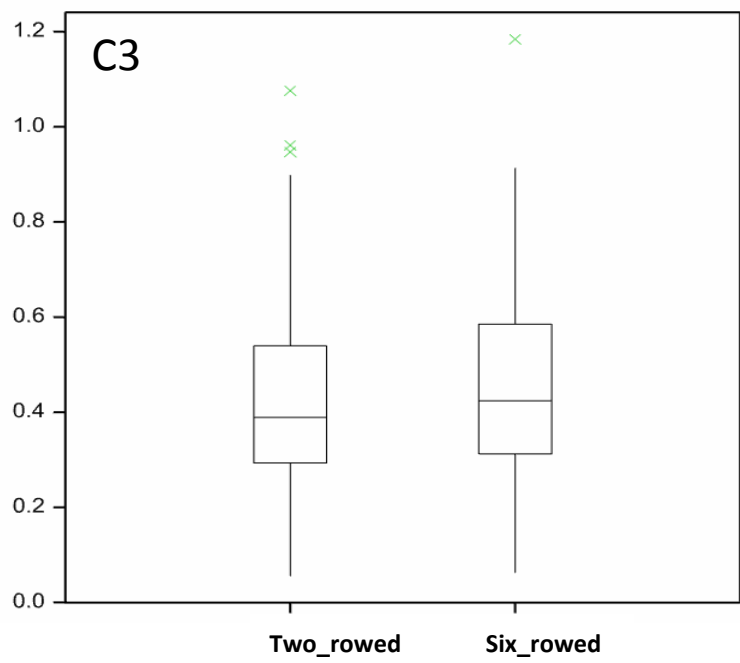

FW DTI

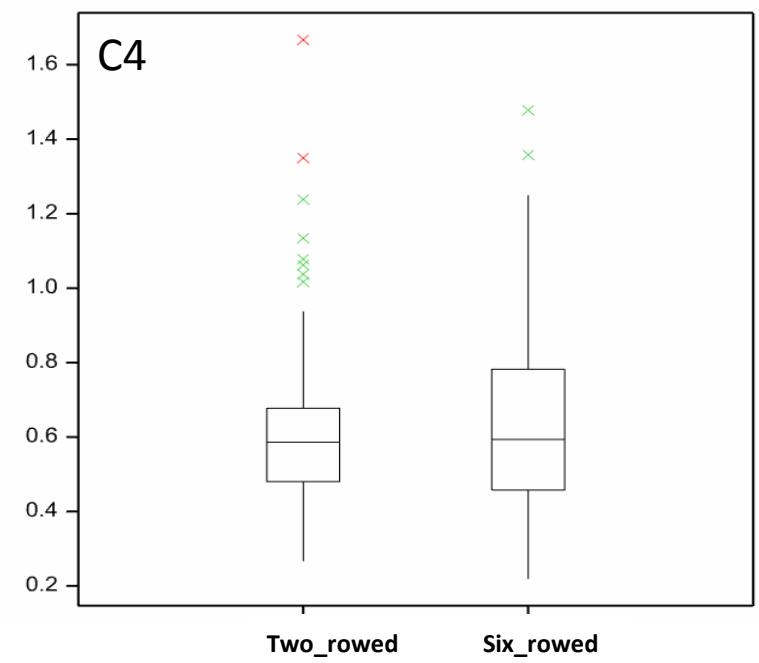

Supplement: S2 Fig — A: Boxplot analysis of variation of the traits based on geographical origin; A1) Germination percentage_DTI, A2) Root Length_ DTI, A3) Shoot Length_ DTI, A4) Fresh Weight_ DTI in barley genotypes. B: Boxplot analysis of variation of the traits based on biological status; B1) Germination percentage_DTI, B2) Root Length_ DTI, B3) Shoot Length_ DTI, B4) Fresh Weight_ DTI in barley genotypes. C: Boxplot analysis of variation of the traits based on row-type; C1) Germination percentage_DTI, C2) Root Length_ DTI, C3) Shoot Length_ DTI, C4) Fresh Weight_ DTI in barley genotypes. (PDF) [file pone.0206682.s005.pdf]
